# Selections and Abstracts

**Published:** 1899-05

**Authors:** 


### SELECTIONS AND ABSTRACTS.

---

### CHLOROSIS.

By DR. S. ASCHER, Hamburg, Germany.

*Translated.*

Although chlorosis in its typical form, which occurs especially in females at the time of puberty, is generally amenable to medical treatment, there are cases in which all our efforts to effect a cure are unattended with successful results. We are inclined in such cases to call to mind the explanation given by Virchow, who assumes that chlorosis frequently depends upon a congenital narrowing of the arteries; yet this explanation is of little aid to the practical physician. If we remember that the action of iron—*our panacea in chlorosis*—is yet a mooted question, and that doubt still exists as to whether iron is capable of absorption by the stomach or intestines, it is natural that we should welcome preparations which promise to give better results than those in previous use.

It is well known that in the hæmoglobin of the red corpuscles *manganese* is constantly found in connection with iron. Opinions have always been divided as to the significance of manganese in the blood, as regards the question whether manganese is really a constant constituent of hæmoglobin or an occasional one. We know that the function of the red corpuscles to take up oxygen is chiefly attributable to the presence of iron, but an active part in this direction has also been ascribed to *manganese.* While in chloride of iron one-third of the chlorine is active, this property belongs to a still greater extent to manganese chloride, a combination of chlorine and manganese corresponding to that of chlorine and iron. Iron chloride is a much more stable combination than manganese chloride, which decomposes even at ordinary temperature and gives off one-half of its chlorine; it is, therefore, *quantitativelg more active than iron. Manganese* as a constituent of the blood exerts a *stronger polarizing effect upon the oxygen* and gives off the latter more readily than iron.

*Manganese is, therefore, a more powerful oxidizing agent than iron,* and absorbed into the body, will exert an energetic assimilative action.

John Kugler, in 1838, was the first to recommend the *manganese* salts in scrofulosis. He made the observation that persons who handled manganese oxide in a chlorine bleachery enjoyed an immunity from disease of the skin, bones and glands. In 1844 Hannan found a diminution of *manganese* in scrofulosis, and to a still greater extent in anemia and chlorosis. In chlorosis he found that the quantity of iron was sometimes chiefly diminished and sometimes that of manganese. *He therefore distinguished chlorosis from lack of iron and manganese.*

Although this schematic classification cannot be accepted, other investigators of more recent times have established a connection between chlorosis and a deficiency of the quantity of *manganese* in the hæmoglobin.

In 1852 Petrequin recommended *manganese* in combination with iron. He maintained that in all cases in which iron is indicated but proves ineffective *there is a deficiency of manganese in the blood.* Among recent authors Rühle, of Bon, has warmly recommended the *combination of manganese with iron* in the treatment of chlorosis, and lately *manganese* has been employed with much success for amenorrhœa in young persons between the ages of eighteen and twenty years.

Notwithstanding these high commendations from various sources, *manganese* was not generally adopted in the treatment of chlorosis, and in cases when iron failed to act resort was had to purely dietetic measures. *The reason for this was that no preparation existed in which iron was combined with manganese in a readily absorbable*

*form.* Such a preparation, however, is Gude's Pepto-Mangan, and the results obtained from its use by myself *and others are exceedingly promising.*

Gude's Pepto-Mangan has been tried by me and a few colleagues in various dieases associated with a depreciated condition of the blood, altogether in *eighty cases,* and in the following I will give a few exact data concerning the observation thus far made by us.

In the simple chlorosis of females during the period of puberty we have employed Gude's Pepto-Mangan in about thirty cases, with uniformly good results. The remedy was always well borne, digestive disturbances were never observed, the marked symptoms of headache, vertigo, palpitation of the heart, and loss of appetite were improved within a few weeks. The bodily weight increased by one-half kilogramme (about one pound). Among the histories of cases at hand the following appear especially noteworthy.

Miss Sched, aged 22, suffered from œdema of the legs, general weakness, marked anemia; menses absent for several years. Prescribed rest, vigorous diet, massage, and Gude's Pepto-Mangan three times daily. After six weeks' treatment œdema disappeared, menses returned, patient felt better, had better color. Four weeks later menses became abundant, although the Pepto-Mangan was no longer employed.

Miss R., aged 28, seamstress, marked anemia, nervous dyspepsia, fluor albus. Besides massage, rest, etc., Gude's Pepto-Mangan, one teaspoonful thrice daily. After three weeks, fluor disappeared, menstruation more abundant, patient's condition perceptibly improved. The disagreeable backache had ceased, appetite and condition of bowels normal.

Miss Clara F., aged 25, weight 52.5 kilogrammes (about 110 pounds); great disturbance of nutrition and anemia; had suffered for five years from amenorrhœa, nervous dyspepsia, general neurasthenia and nervousness; complexion sallow, owing to constipation. Gude's Pepto-Mangan administered (altogether 1,100 grammes, 36 to 37 ounces). Result very favorable; weight increased one-half kilogramme (about one pound) every week, appearance excellent, general condition much improved; constipation relieved by extract frangul. fluid. During the eighth week menses returned; headache and stomach troubles have disappeared; patient has great hopes of restoration to perfect health.

This preparation also proved very serviceable in cases of anemia associated with more or less marked scrofulosis. The abscesses of the skin healed, eczema, of undoubted scrofulous character, disappeared. The following case is characteristic:

Margaret G., aged 12; a weak, anemic, and scrofulous girl, had suffered repeatedly from tonsillitis, coryza, anorexia, glandular swellings, and had a pale and sickly appearance. Prescribed for a period of six months three baths containing Kreuznach mother-lye thrice weekly, and Gude's Pepto-Mangan one teaspoonful thrice daily. In all 1,000 grammes (two pounds) of the liquor were used. The girl now looks well, healthy complexion, red cheeks and lips, appetite good, swelling of glands has almost entirely disappeared.

I have further employed Gude's Pepto-Mangan in that form of anemia which is found in young women as a complication of uterine trouble or as a consequence of profuse loss of blood from repeated abortions or childbirths. The effect was always uniformly good, The patients, who belonged for the most part to the working class. after three to four weeks' use of the Pepto-Mangan, were able to resume work (although their nutrition could only be slightly improved), and were able to accomplish as much as formerly.

It is well known that during the course of chronic malaria marked anemia develops, which is extremely obstinate to treatment and frequently defeats all efforts to effect a cure. Even after the attacks of fever have subsided the anemia quite often persists for a long time, and the patient becomes greatly reduced in health.

In this condition, where, as I have said, other preparations of iron frequently leave us in the lurch, Gude's Pepto-Mangan has rendered us good service. We have had occasion to employ this remedy sixteen times in anemia following malaria, and report the following two cases by way of illustration:

Margaret Sch., aged 26; unmarried, scrofulous tumors of the neck, anemia following malaria, gastric catarrh; bodily weight 58 kilogrammes (about 122 pounds). Duration of treatment two months; 800 grammes of Pepto-Mangan used with material and continuous improvement. Vomiting and headache have disappeared, appetite good, increase of weight two kilogrammes (four pounds).

Bertha Pr., aged 10 years; 20.5 kilogrammes (about 43 pounds), marked anemia after malaria and scarlatina, diphtheria. Five hundred grammes (one pint) of Gude's Pepto-Mangan administered in six weeks. Considerable improvement of the general condition. The patient had so much improved that treatment was discontinued, thinking it no longer necessary. Increase of weight 1.5 kilogrammes (three pounds).

That Gude's Pepto-Mangan is also an excellent remedy for children is demonstrated by the above observation, as well as the following one:

Annie and Willie D., twins, 2¾ years old. Rickety, pale and unhealthy color of face, appetite poor. Gude's Pepto-Mangan in wine, one teaspoonful thrice daily, altogether 300 grammes (ten ounces) used. The children take it gladly and it is well borne. Appetite has improved.

Finally, it may be mentioned that I have tried the Pepto-Mangan in several cases of pulmonary tuberculosis. Of course, the effect here was only relative, yet frequently we were able to improve the appetite and effect a slight gain in weight.

In the foregoing remarks I have somewhat in detail given my experience with Gude's Pepto-Mangan, and I have done this because I am convinced that it is worth while to institute further trials with this preparation. The observations thus far made were very encouraging. I will not attempt to define what part manganese plays in the new preparation. At any rate it appears that, compared with other ferruginous preparations, Gude's Pepto-Mangan has a better and more certain effect, and is characterized by the fact that it does not produce disturbance of the digestive tract. It would be interesting to determine by experimentation that under the use of this remedy the quantity of manganese in the blood is actually increased. Such an experiment would definitely prove that Hannan's theory of chlorosis based upon deficiency of iron and manganese in the blood is perfectly correct.—*From the Allgemeine Medizin. Central Zeitung.*

### ACTION OF ALCOHOL ON THE RESPIRATORY CENTER.

Wilmanns.—Direct excitation of the respiratory center by alcohol (*Pflueger's Archiv.*, v. 66, p. 167). Boriz's observation, that injection of moderate quantities of alcohol into the veins of rabbits increases markedly the respiratory volume, has been corroborated by Jaquet, who extended the observation and found that the same occurred after its administration per os. Jaquet believed it was due to reflex, and not a direct action upon the respiratory center. He based this view upon the fact that the post-mortem examination showed an intense gastric irritation, and that by irritation of the nerves of the gastric mucous membrane the respiratory volume was increased, and after breathing alcohol vapors there was likewise an increase in respiratory volume, which did not occur if the vagus was previously cut, and finally after the administration of 0.002 g. morphine, which he believed lessened peripheral irritability, there was no increase in volume. Wilmanns questions Jaquet's results, and believes it due to a central irritation, because the gastric mucous membrane of his animals was not reddened more than normal, and irritation of the gastric mucous membrane with mustard oil did not increase their volume. Contrary to Jaquet, breathing alcohol vapors after cutting the vagi still increased them, and in spite of the morphine the respiratory volume was still increased. This amount of morphine, 0.002, Wilmanns holds, influences the repiratory center, and, finally, alcohol acts quicker if injected directly into the carotid artery than when into a vein. These observations give a pharmacological basis to the clinical use of alcohol.—*The Dominion Medical Monthly.*

---

### CHLOROFORM FOR TAPEWORMS.

Carratù.—Use of chloroform for tænia. (*Giorn. med.*, 1897, Nos. 8 and 9.) Carratù has used chloroform in many cases as an anthelmintic; he recommends it for its prompt action and its almost entire freedom from untoward action. He uses it as follows:

| | |
|---|---|
| Chloroform | 3—4 |
| Syrup | 35 |

One teaspoonful every two hours, beginning early in the morning, and one hour after the last dose, 25—30 gm. castor-oil is given, the patient being on a bland diet. He claims to have cured thus cases which had resisted the action of felix mas, kousso, etc.—*The Dominion Medical Monthly.*

